# Cognitive processing load during listening is reduced more by decreasing voice similarity than by increasing spatial separation between target and masker speech

**DOI:** 10.3389/fnins.2014.00088

**Published:** 2014-04-29

**Authors:** Adriana A. Zekveld, Mary Rudner, Sophia E. Kramer, Johannes Lyzenga, Jerker Rönnberg

**Affiliations:** ^1^Department of Behavioural Sciences and Learning, Linköping UniversityLinköping, Sweden; ^2^Linnaeus Centre for Hearing and Deafness Research, The Swedish Institute for Disability Research, Linköping and Örebro UniversitiesLinköping, Sweden; ^3^Section Audiology, Department of Otolaryngology-Head and Neck Surgery and EMGO Institute for Health and Care Research, VU University Medical CenterAmsterdam, Netherlands

**Keywords:** speech perception, pupil response, spatial cues, voice cues, interfering speech, cognitive abilities

## Abstract

We investigated changes in speech recognition and cognitive processing load due to the masking release attributable to decreasing similarity between target and masker speech. This was achieved by using masker voices with either the same (female) gender as the target speech or different gender (male) and/or by spatially separating the target and masker speech using HRTFs. We assessed the relation between the signal-to-noise ratio required for 50% sentence intelligibility, the pupil response and cognitive abilities. We hypothesized that the pupil response, a measure of cognitive processing load, would be larger for co-located maskers and for same-gender compared to different-gender maskers. We further expected that better cognitive abilities would be associated with better speech perception and larger pupil responses as the allocation of larger capacity may result in more intense mental processing. In line with previous studies, the performance benefit from different-gender compared to same-gender maskers was larger for co-located masker signals. The performance benefit of spatially-separated maskers was larger for same-gender maskers. The pupil response was larger for same-gender than for different-gender maskers, but was not reduced by spatial separation. We observed associations between better perception performance and better working memory, better information updating, and better executive abilities when applying no corrections for multiple comparisons. The pupil response was not associated with cognitive abilities. Thus, although both gender and location differences between target and masker facilitate speech perception, only gender differences lower cognitive processing load. Presenting a more dissimilar masker may facilitate target-masker separation at a later (cognitive) processing stage than increasing the spatial separation between the target and masker. The pupil response provides information about speech perception that complements intelligibility data.

## Introduction

When speech perception is challenged by interfering speech signals, listening depends on both auditory factors and cognitive abilities like working memory capacity (Rönnberg, 2003; Rönnberg et al., 2013). The accumulating evidence for the role of cognitive abilities in speech perception (for reviews, see Akeroyd, 2008 and Besser et al., 2013 and see also Rönnberg, 2003; Kramer et al., 2009; Rönnberg et al., 2013) has resulted in an increase in research focused on the measurement of cognitive processing load during listening (Rabbitt, 1968; Rakerd et al., 1996; Gosselin and Gagné, 2011; Mackersie and Cones, 2011; Picou et al., 2011; Wild et al., 2012; Mishra et al., 2013a). In the present study, we applied pupillometry to assess cognitive processing load. The pupil size increases with increasing cognitive processing load induced by increasing task demands (e.g., Beatty, 1982; Engelhardt et al., 2010), including intelligibility level (Zekveld et al., 2010), sentence complexity (Piquado et al., 2010), visual context (Engelhardt et al., 2010), lexical competition (Kuchinsky et al., 2013) and masker type (Koelewijn et al., 2012a). Larger working memory capacity and better linguistic closure ability are associated with larger pupil dilation amplitude and a longer peak latency of the pupil response (Zekveld et al., 2011; Koelewijn et al., 2012b; Zekveld and Kramer, 2014), indicating that the allocation of larger amounts of cognitive capacity may come with more intensive mental processing in more difficult listening conditions (Ahern and Beatty, 1979; Van der Meer et al., 2010; Grady, 2012; Koelewijn et al., 2012b; Ng et al., 2013). Importantly, the cognitive processing load evoked by speech perception can be dissociated from the actual speech perception performance, as cognitive processing load can vary in conditions in which speech perception performance is similar (Mackersie and Cones, 2011; Koelewijn et al., 2012a).

The perception of speech in interfering sounds can be aided by different types of acoustic cues. For example, when female speech maskers are used for female target speech, talker-specific voice cues (e.g., voice-related pitch cues) distinguishing target and masker are less salient than when male speech maskers are used for female target speech. Less salient speech segregation cues generally result in reduced ability to perceive the target speech (Brungart et al., 2001). Additionally, if the target speech and interfering sounds come from different spatial locations, the speech reception thresholds (SRTs; the signal-to-noise ratio [SNR] required for a certain level of speech perception performance) of listeners with normal hearing can improve by as much as 18 dB SNR, depending on the amount of spatial separation between the sounds (Arbogast et al., 2002, 2005; Cameron et al., 2011). This benefit is referred to as spatial release from masking. The spatial release from masking is larger when the acoustic characteristics of the masker are more similar to those of the target speech (Arbogast et al., 2005; Best et al., 2012). The aim of the present study was to investigate the influence of target-masker similarity (i.e., differences in gender and spatial origin between the target and masker voices, and the interaction between these signal characteristics) on cognitive processing load indexed by the pupil response. We also studied the relation between individual differences in cognitive abilities, speech perception performance and the pupil response in different conditions.

Despite the fact that the relevance of cognitive abilities in speech perception has increasingly been acknowledged in the past decades (for a review, see Arlinger et al., 2009), only a few studies have assessed the role of cognitive abilities in spatially complex listening conditions. These studies (e.g., Neher et al., 2009, 2012; Glyde et al., 2013) suggest that better cognitive abilities are associated with better speech perception performances. The relation tended to be stronger when verbal measures of working memory are applied as compared to a more general cognitive screening instrument (Cognistat; Mueller et al., 2001) that measured eight cognitive functions (including attention, memory and language) with the aim of identifying cognitive deficits (Neher et al., 2009, 2012; Glyde et al., 2013). Also, the association was stronger when the origin of the maskers differed from that of the target speech as compared to co-located speech and maskers (Neher et al., 2009). Neher et al. (2009) argued that for the co-located target and masker condition presented in their study, listeners could basically only rely on level cues to segregate target and maskers. Consequently, performance was limited by the accessibility of auditory cues rather than top-down abilities. They also suggested that the relatively large amount of *“mental effort”* required to parse the target speech at the negative SNRs applied in the conditions with spatially separated target and masker speech could have driven the cognitive involvement in that condition. Similarly, Best et al. (2012) suggested that cognitive abilities play a larger role in speech perception when SNRs are negative. Gatehouse et al. (2003) also argued that it is important to take into account possible interactions between signal characteristics and cognitive abilities. These previous studies indicate that individual differences in cognitive abilities interact with the characteristics of the target and masker. It would be interesting to examine whether objective measures of *cognitive processing load* also reflect variations in target-masker similarity. For example, if spatial separation between the target and masker signals reduced cognitive processing load even when intelligibility levels were equalized, this would demonstrate an additional benefit of spatial cues that is not reflected by intelligibility data.

To our knowledge, no previous study has investigated the effect of voice characteristics and location differences between target and masker speech on the pupil response during listening. In the present study, we measured the pupil dilation response to listening to female speech masked by speech from either female or male speakers. Listeners rely on any differences in the characteristics of the voices (e.g., voice saliency or distinctiveness) to distinguish the target and masker voices, including level differences and a priori knowledge of the target voice characteristics (Brungart, 2001; Brungart et al., 2001). The method applied in the present study is similar to that of the LISN-S test (Cameron and Dillon, 2007). LISN-S measures the benefits due to voice and spatial cues, separately and combined. In the present study, we aimed to assess the influence of voice cues (female vs. male maskers) and spatial cues on speech perception performance and the pupil response. In a two- by two design giving four conditions, the similarity of the target voice and interfering speech maskers was varied, as well as the spatial separation between the masker and the target speech. We used HRTFs to manipulate the virtual spatial location of two streams of masker speech: these were perceived either from the same location as the target speech (0° azimuth) or from + and −90° (±90: one stream from the left of the listeners, and one from the right).

Furthermore, we assessed a range of cognitive functions known to be associated with speech perception performance when the listening takes place under adverse conditions (Kramer et al., 2009; Koelewijn et al., 2012a; Besser et al., 2013; Ellis and Munro, 2013) and the pupil response during listening to speech in background maskers (see Koelewijn et al., 2012b; Zekveld and Kramer, 2014). These were: working memory capacity (the reading span test [RSpan, Daneman and Carpenter, 1980; Rönnberg et al., 1989, 2013]) and the size comparison test [SicSpan, Sörqvist et al., 2010], information updating (the letter memory test; Morris and Jones, 1990), the ability to perceive degraded linguistic information [text reception threshold test (TRT, Zekveld et al., 2007)] and executive control abilities [the trail making test (Reitan, 1958)].

We expected, in line with the results of Neher et al. (2009) and Glyde et al. (2013), that better cognitive abilities would be associated with better speech perception. Also consistent with their findings, we expected this association to be strongest when cues distinguishing target from masker were maximized, that is when different-gender masker voices originated from a location different from that of the target. In these conditions, cognitive abilities can be used to benefit from the available cues. We expected that the pupil response would be larger with fewer voice and spatial cues available, as in these conditions, it is harder to segregate target speech from noise.

### General methods

The test session started with pure-tone audiometry and near vision screening. Then, the reading span test (verbal working memory capacity) was presented. Participants performed a practice speech perception test, followed by the first speech perception block. In the speech perception tests, we employed a two-factor within-subjects factorial design, crossing two masker voices (male or female) with two spatial configurations (masker speech from 0° or ±90°). Then, participants performed the SicSpan test (verbal working memory capacity and inhibition), followed by a break, a second practice test and the second speech perception block. Subsequently, participants performed a practice TRT test and three additional TRT tests (linguistic closure). The test session was finished after performing the letter memory (information updating) and trail making (executive control ability) tests. The duration of the test session was 1.5 h with a 5-min-break halfway through the test session. The rationale for presenting two different tests of verbal working memory was that previous studies have shown that each of those tests can be differentially associated with speech perception performance and/or the pupil response evoked by different conditions (Koelewijn et al., 2012b; Sörqvist and Rönnberg, 2012; Besser et al., 2013).

### Participants

Twenty-four young adults [20 women, 4 men; mean age 22 yrs, standard deviation (SD) = 2.8 yrs] with normal hearing thresholds participated. Flyers and advertisements were used to recruit students and employees of VU University and VU University Medical Centre. All participants were native Dutch speakers and had normal or corrected-to-normal vision as screened with a near vision test (Bailey and Lovie, 1980). Pure-tone hearing thresholds of the participants were measured to ensure that the thresholds of both ears were ≤20 dB HL at the octave frequencies between 125 and 8000 Hz. All participants had normal hearing thresholds; the mean pure-tone hearing thresholds were on average 7.2 dB HL (*SD* = 7.4 dB). The exclusion criteria were the following: dyslexia or other reading problems, or a history of a neurological or psychiatric disease. The project was approved by the Ethics Committee of the VU University Medical Center. All participants provided written informed consent.

### Stimuli

The target and masker stimuli were selected from the meaningful, semantically neutral sentence material developed by Versfeld et al. (2000) and recorded with a sampling rate of 44100 Hz and a bit depth of 16 bits. Each sentence contained eight to nine syllables and no word contained more than three syllables. The individual words in the sentences were articulated at an average rate of 3.4 words per second across all sentences. An example sentence (translated into English) is: “the shop is within walking distance” (Versfeld et al., 2000). The target sentences were pronounced by a female speaker, and were always perceived from the front (0° azimuth) of the listener. The masker consisted of two independent streams of concatenated sentences that were played continuously, back-to-back, without silent gaps between the sentences. The onsets of target and masker sentences were not coordinated in time; the masker speech streams could start in the middle of a sentence. The two streams of masker speech were always from the same talker who was either male or female. The mean and range of the duration of the target sentences did not differ from that of the female and male masker sentences. On average, the mean sentence duration was 1.9 s, ranging from 1.3 to 3.0 s. The onset of the target sentence occurred 3000 ms after masker onset and target sentence offset was 4000 ms before masker offset. This allowed the measurement of the pupil response to masked speech while preventing the onset and offset of the masker stimulus from influencing the pupil dilation response between target-speech onset and the response of the listeners. The overall intensity of the target-masker mixture was fixed at 70 dB SPL; the SNR was varied by adapting both the level of the target speech and the level of the maskers.

Virtual target/masker separation (+90 and −90° azimuth; one stream from the left and the other from the right) and co-location (0° azimuth) were achieved using HRTFs that were developed using the KEMAR mannequin with the large pinnae (Algazi et al., 2001). We used the left-ear HRTFs in our tests, and used the mirror image of the left ear HRTFs for the right ear. Using HRTFs to manipulate the perceived location of sounds alters their frequency spectrum, therefore the spectrum of the masker speech will differ for presentation from 0 and ±90° azimuth. Such spectral differences may affect speech reception scores as indicated by the Speech-Intelligibility Index SII (ANSI, 1997). To prevent this, the long-term average frequency spectrums of the male and female masker speech in the 0-degree configuration were shaped using finite impulse response filtering to match those of the corresponding, combined, maskers from the +90 and −90° directions, in order to prevent any spectral differences between the masking stimuli from confounding the effects of spatial configuration on speech reception scores and pupil responses. The novel signals had a slightly different timbre and were evaluated by listening to them; no artifacts or changes in perceived location were observed. Prior to data collection, a pilot test was performed in which we asked five subjects to indicate the direction of the sound sources and evaluate the quality of the signals. The results indicated that the manipulation served its purposes and no further changes were required.

### Set-up

Test administration took place in a sound-attenuated room. The audiogram was made using an audiometer (Decos Systems B.V., software version 2010.2.6) connected to TDH 39 headphones. Auditory stimuli in the experimental tests were presented by an external soundcard (Creative Sound Blaster Audigy) through Sony MDR V900 headphones (Sony Corporation). Subjects were seated behind a SMI iView X RED remote eye-tracking system with spatial resolution of 0.03° and sampling frequency of 60 Hz. A PC screen was positioned on top of the pupillometric system, about 45 cm away from the subject's head. Subjects focused on a fixation dot presented in the middle of the screen.

### Procedure

In four conditions (2 masker voices × 2 spatial configurations), the SNR required for 50% correct sentence perception was estimated using an adaptive procedure. This entailed changing the SNR for each sentence, based on the response to the previous sentence. The SNR of a sentence dropped by −2 dB following a single correct response, and increased by 2 dB following a single incorrect response. The SNR of the first sentence was −4 dB for the 0 degree condition and −10 dB for the ±90° condition. Subjects were asked to repeat the sentences aloud. They were instructed to wait until after masker offset (4 s after target speech offset) to make their response. The experimenter scored their answers. A sentence was scored correct if all words of the sentence were repeated in the correct order. In each condition, a list of 25 sentences was presented, as this allows a reliable estimation of the pupil response. The 25 sentences were randomly selected from 2 phonemically-balanced lists of 13 sentences created by Versfeld et al. (2000). The adaptive procedure resulted in a sentence intelligibility level of approximately 50% correct in each of the conditions. However, the SRTs (i.e., the average SNR of sentences 5–25) differed between the conditions. The rationale for this approach was that intelligibility differences have a large effect on the pupil response (Zekveld et al., 2010). Therefore, intelligibility should be controlled for when assessing the influence of other factors, such as masker characteristics. SNR differences itself are unlikely to have a major influence on the pupil response. For example, Koelewijn et al. (2012a) showed that stationary and fluctuating noise maskers evoked similar pupil dilation responses despite relatively large differences in SRT when sentence intelligibility was the same for the two maskers.

SRT testing was blocked by masker voice. Within blocks, the order of sentences from each of the two conditions (two spatial configurations) was pseudo-randomized with the restrictions that no more than two sentences from the same condition should be presented sequentially and that the difference in the cumulative number of sentences per condition should not exceed two at any point in the test block. This ensured that the procedures ran approximately in parallel, preventing any confounding order effects on performance or the pupil response. The order of masker voice blocks was counterbalanced across participants. The allocation of sentence lists to conditions was also counterbalanced across participants.

### Pupillometry

The location and size of the pupil of the left eye were measured during each target-masker presentation (trial). Before the experiment started, the pupil size was measured in maximum illumination (100 lx) and in complete darkness. The room illumination was adapted individually such that the pupil size was around the middle of its dynamic range at the start of the experiment. This prevents ceiling and floor effects in the pupil response and makes the response independent of the baseline pupil size (Beatty and Lucero-Wagoner, 2000). The mean room illumination after individual adjustments across participants was 51 lux (*SD* = 24 lux).

The baseline pupil size in each trial was defined as the average pupil size during the first 1.0 s of the presentation of the masker, (between 3 s and 2 s prior to target-speech onset). The mean pupil diameter in each trial was calculated by averaging the pupil size between target speech onset and masker offset for the shortest sentence in the set (i.e., 5.3 s after target speech onset). Pupil diameters below 3 standard deviations of the mean diameter of each trial were coded as a blink. If the data contained more than 15% blinks between the start of the baseline and masker offset, the trial was excluded from data analysis. The pupil data were furthermore visually inspected for artifacts due to eye-movements. The pupil data for the first trial in each block were omitted from the analysis, as the adaptive SRT procedure commenced during this sentence. On average, the pupil data of 21 trials were included in each condition. Eye-blinks were replaced by linear interpolation starting 4 samples before and ending 8 samples after a blink. A 5-point moving average smoothing filter was passed over the selected and deblinked pupil data. Per trial, we determined the *peak pupil dilation* (peak dilation amplitude in mm) relative to the baseline pupil size in the same trial. Finally, the peak pupil dilation was averaged over trials, separately for each participant and condition.

### Tests assessing cognitive abilities

#### Text reception threshold test

The TRT test measures the ability to perceive masked linguistic (text) information, also called “linguistic closure” ability (Besser et al., 2013). A total of 13 printed sentences (Versfeld et al., 2000) masked by a bar pattern were presented on a PC screen (see Zekveld et al., 2007). The sentences were different from those presented in SRT tests. The field background color was white, text color was red, and the color of the mask was black. At the start of each trial, the masker appeared with the text “behind” it in a word-by-word fashion. Display-onset of each word in the sentence was equal to the timing of the start of the utterance of each word in the corresponding audio file (Versfeld et al., 2000). The average duration of the audio utterance of the words was 281 ms, ranging from 44 to 854 ms. All words remained on the screen for 3500 ms after completion of the sentence. Participants were asked to read the sentences out loud. The experimenter scored whether the sentences were read entirely correctly. The masking percentage of the first sentence was 58% unmasked text. A 1-up-1-down adaptive procedure with a step-size of 6% was applied, targeting the percentage of unmasked text required to read 50% of the sentences correctly. The TRT was the average proportion of unmasked text for sentences 5–14; lower TRTs indicate better performance. The fourteenth sentence was not actually presented. However, the percentage of unmasked text for this sentence followed directly from the response to the previous sentence. We included this value in the calculation of the TRT to obtain a better estimate of the threshold (Plomp and Mimpen, 1979). Participants performed one practice and three regular TRT tests, and we used the TRT averaged over the three tests in the analysis.

#### Reading span test

The RSpan test (Daneman and Carpenter, 1980) measures verbal working memory capacity. In this test, 5-word Dutch sentences were presented visually. The materials were developed (Besser et al., 2013) to be equivalent to the Swedish version described by Rönnberg et al. (1989) and Andersson et al. (2001), in turn based on an English version (Baddeley et al., 1985). Half of the sentences are semantically incoherent (e.g., “The table sings a song.”) and half are coherent (e.g., “The friend told a story”). First, three sets of three sentences were presented, followed by three sets of four sentences, three sets of five sentences, and three sets of six sentences. After each sentence, participants verbally indicated whether the sentence made sense or not. After each set of sentences, participants were asked to orally recall all first or all last nouns of the sentences in the set in serial order. The experimenter recorded the total number of words correctly recalled regardless of order. The maximum total score is 54.

#### Size comparison span

The size-comparison span (SicSpan) task (Sörqvist et al., 2010) measures verbal working memory capacity and also examines the ability to suppress irrelevant information. Sets of size-comparison questions like “is a BUSH larger than a TREE?” were presented on a PC screen. Then, a semantically related and to-be-remembered word like FLOWER was presented. Ten sets were presented in total; the set sizes ranged from 2 to 6 with each set size being presented twice. Within sets, nouns used in the questions and those to be remembered were from the same semantic category, but between sets these categories differed. Immediately after each question, participants responded to the question by pressing one of two buttons corresponding to “yes” or “no.” After each set participants were asked to orally recall the to-be-remembered items. The SicSpan score was the total number of correctly recalled items regardless of order (maximum of 40), with higher scores reflecting better performance.

#### Letter memory test

To assess information updating, the visual letter memory task (Morris and Jones, 1990) was applied. A series of 5, 7, 9, or11 letters (consonants) was presented visually at the center of the screen for 2 s each using a DMDX platform (Forster and Forster, 2003). Each sequence length was presented three times, and the order of the sequence lengths presented was randomized. Two lists consisting of 7 and 9 letters each were presented as practice tests. Twelve lists were used in total. The participants were told that the presentation would end unexpectedly. They were asked to recall, in any order, the last four items presented. The total number of correctly recalled letters was scored (maximum score = 48).

#### Trail making

The trail making test (Reitan, 1958) consists of two parts. Part A is sensitive to visuo-perceptual abilities, and part B reflects working memory and task-switching ability. The difference in reaction times between the two parts (B–A) represents executive control abilities (Sánchez-Cubillo et al., 2009). In part A, a sheet of paper with 25 encircled numbers (1–25) was presented to the participant. In part B, a sheet of paper with 12 numbers (1–12) and 12 letters (A–L) was presented. For part A, participants had to draw lines sequentially connecting the numbers and for part B, they had to draw lines alternating between numbers and letters (e.g., 1, A, 2, B, 3, C, etc.). The amount of time required to complete each part was measured. We assume that control abilities are relevant for speech perception in the current study, because listeners need to focus on and follow the target speech while ignoring speech from two masker voices. Therefore, we used the B-A difference measure in the correlation analysis. This measure will be referred to as Trail-dif.

### Statistical analyses

We assessed the influence of masker voice (male, female) and spatial configuration (0°, ±90°) on the SRTs in the adaptive conditions using repeated-measures analyses of variance (ANOVA). Repeated measures ANOVA with the same factors was also performed on the peak pupil dilation. Finally, we performed a correlation analysis to assess the strength of the associations between the TRT, RSpan, SicSpan, letter memory and Trail-dif performances on the one hand and the SRTs and peak pupil dilation amplitudes during the SRT tests on the other hand. We did not make adjustments for multiple comparisons in this correlation analysis.

## Results

### Descriptive statistics: cognitive tests

The descriptive statistics of the performances on the cognitive tests are presented in Table 1. The range in scores on the cognitive tests was comparable to that observed in other studies with similar subject groups (e.g., Zekveld et al., 2007; Besser et al., 2012, 2013; Mishra et al., 2013b; Zekveld and Kramer, 2014).

**Table 1:** **Mean, standard deviation, and range of the performances on the cognitive tests**.

	**Mean**	***SD***	**Range (maximum score)**
Reading span	21.7	5.4	12–34 (54)
Size comparison span	29.8	6.7	13–38 (40)
Text reception threshold	53.6%	2.9%	47.8–59.8%
Letter memory	41.6	3.8	35–47 (48)
Trail A	18.1 s	5.3 s	11.5–30.8 s
Trail B	37.3 s	16.9 s	20.3–83.9 s
Trail-dif	19.2 s	14.7 s	5.3–57.7 s

*The maximum score on each test is indicated between parentheses*.

### Speech perception test results

**Figure 1:**
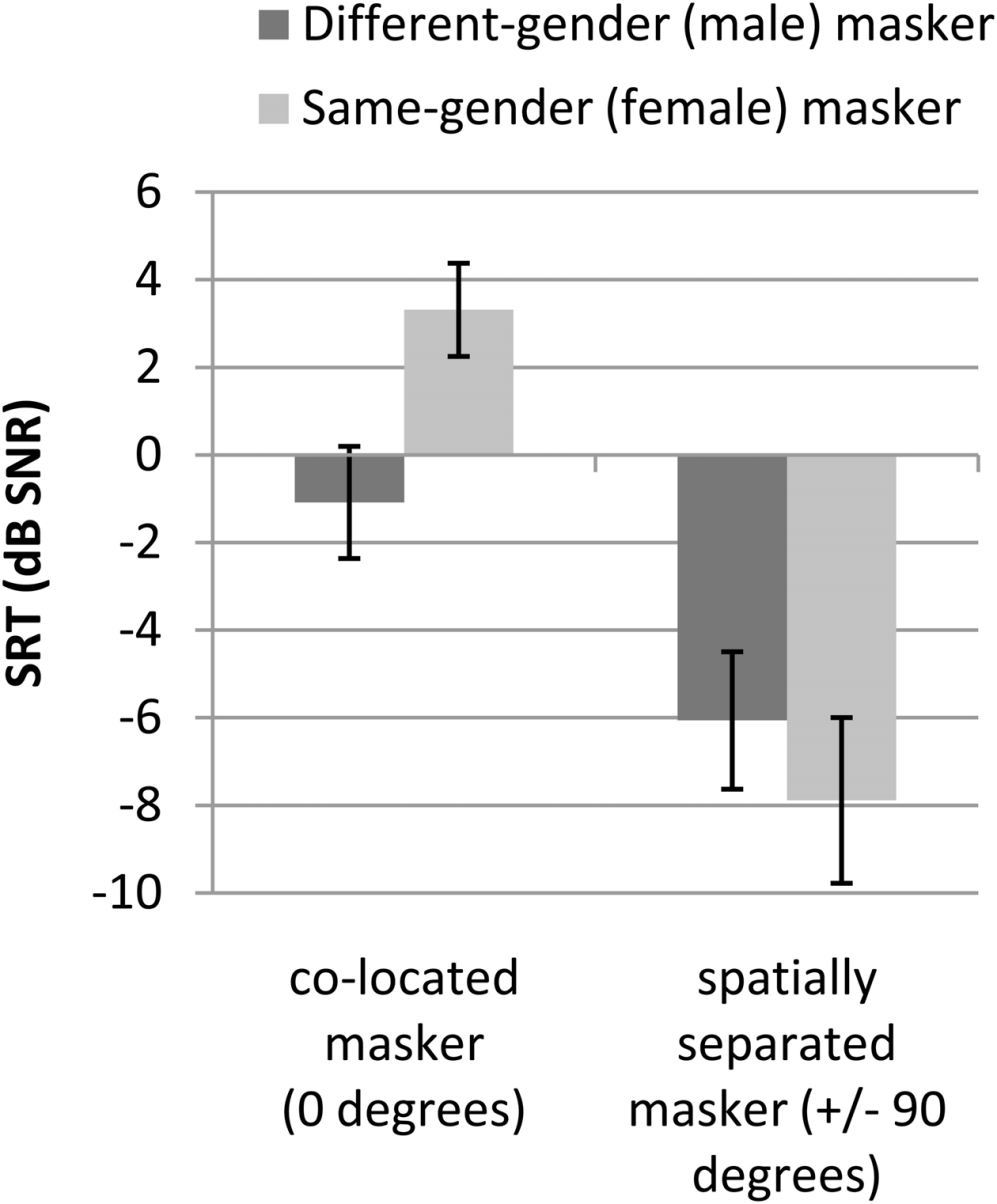
**Average speech reception thresholds (SRT) in dB signal-to-noise ratio (SNR)**. Error bars reflect standard deviations. Twenty-four participants were tested.

The behavioral speech perception performance data are shown in Figure 1. The Figure shows that the estimated SNR required for 50% sentence perception thresholds is higher (worse) for the co-located (0 degree) as compared to the spatially separated (±90°) conditions. It also shows that the threshold is higher for the same-gender (female) as compared to the different-gender (male) masker in the 0° conditions, but that the threshold is higher for the different-gender as compared to the same-gender masker in the ±90° condition.

The repeated-measures ANOVA on the SRTs with independent variables masker voice (male, female), and spatial configuration (0°, ±90°) revealed a main effect of masker voice, such that estimated thresholds were lower (better) for the different-gender compared to the same-gender masker [*F*_(1, 23)_ = 23.7, *p* < 0.001]. The ANOVA also showed a main effect of spatial configuration, with lower thresholds in the spatially separated than in the co-located conditions [*F*_(1, 23)_ = 573.0, *p* < 0.001]. An interaction effect between masker voice and perceived spatial location was observed as well [*F*_(1, 23)_ = 194.2, *p* < 0.001]. *Post-hoc* paired *t*-tests indicated that for both the male and the female maskers, the differences in SRTs between the 0 and ±90° configuration were statistically significant [*t*_(23)_ = 13.9, Bonferroni corrected *p* < 0.00001 and *t*_(23)_ = 25.0, Bonferroni corrected *p* < 0.00001, respectively]. For both the 0° and ±90° conditions, the difference in SRTs between the male and female maskers was statistically significant [*t*_(23)_ = 14.9, Bonferroni corrected *p* < 0.00001 and *t*_(23)_ = 4.7, Bonferroni corrected *p* = 0.0004, respectively]. The interaction effect indicates that the effect of different-gender maskers, as compared to same-gender maskers, is larger for co-located target speech and maskers and that the effect of spatial separation is larger for same-gender maskers.

### Results pupillometry

**Figure 2:**
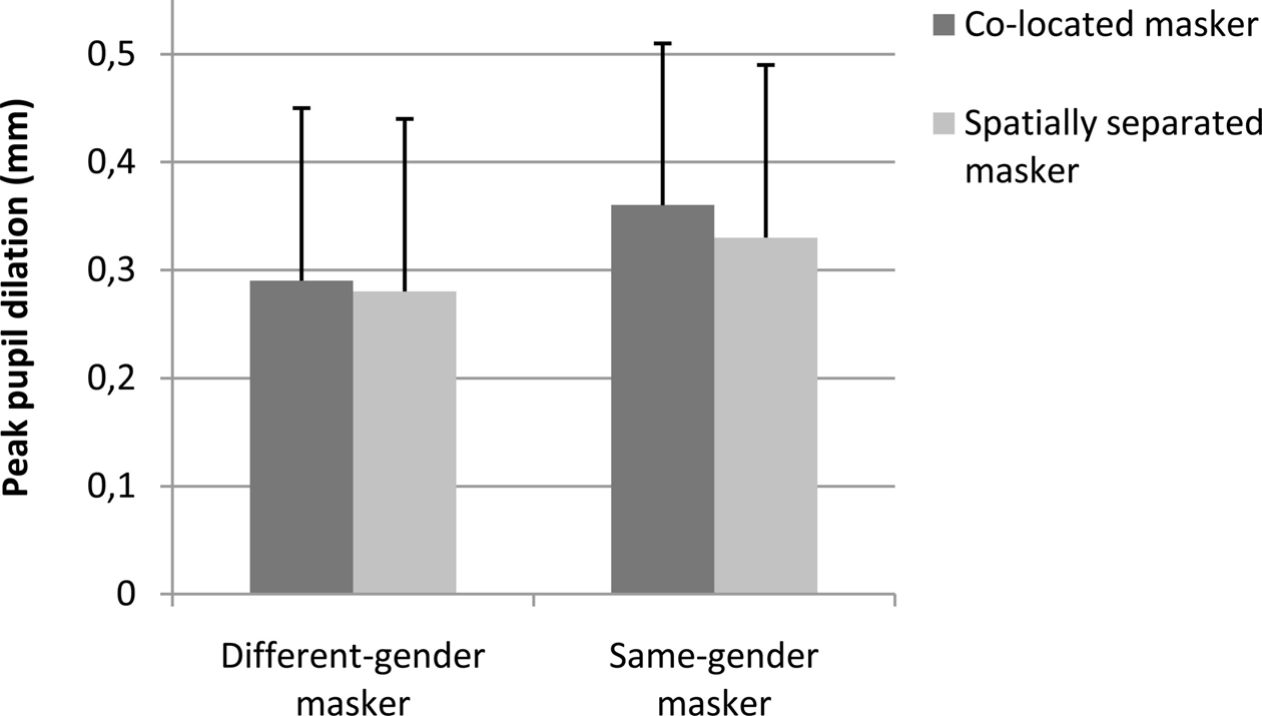
**Peak dilation amplitude of the pupil response during speech perception**. Error bars reflect standard deviations. The pupil dilation is calculated relative to the baseline pupil size in the interval between 3 s and 2 s prior to the onset of the target speech. The peak dilation amplitude was the maximum pupil size in the interval between target speech onset and masker offset for the shortest sentence in the set (i.e., 5.3 s after target speech onset). Twenty-four participants were tested.

**Figure 3:**
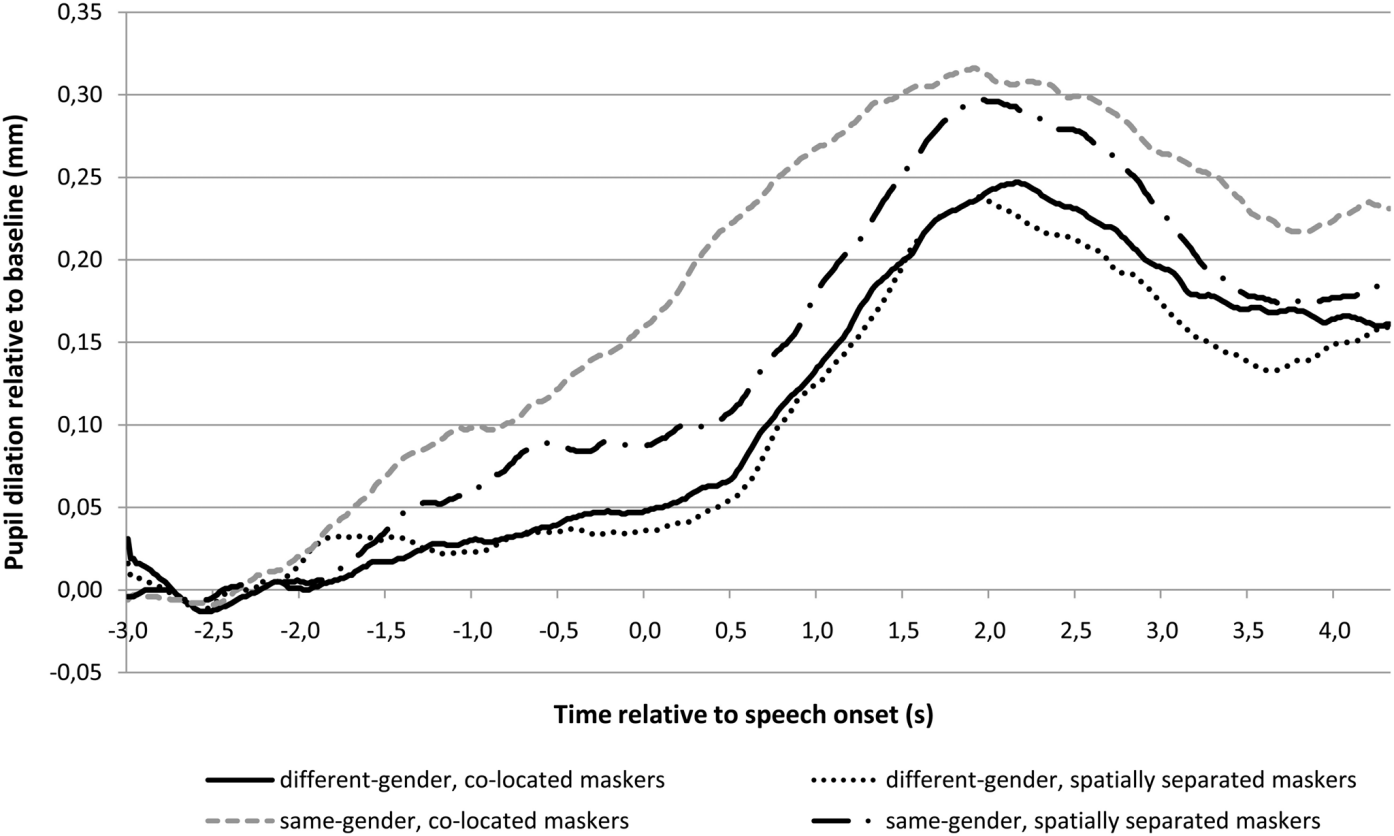
**Pupil response in the four speech reception threshold conditions as function of time relative to the onset of the target speech (time 0 s)**. The pupil dilation is calculated relative to the baseline pupil size in the interval between 3 s and 2 s prior to the onset of the target speech. Twenty-four participants were tested.

Figures 2, 3 show the pupil response, in average peak amplitude and the time course of it, respectively. Table 2 shows the baseline pupil size and peak pupil dilation in each of the four conditions. As shown in Figures 2, 3, the pupil dilation response was largest for the condition with same-gender masker and no spatial separation, followed by the condition with same-gender masker and spatial separation, and we observed smaller pupil responses for the conditions with different-gender maskers.

**Table 2:** **Mean peak dilation amplitude (mm) and baseline pupil size (mm) in each of the 4 conditions**.

**Procedure**	**Different-gender (male) masker**		**Same-gender (female) masker**	
	**Co-located masker**	**Spatially separated masker**	**Co-located masker**	**Spatially separated masker**
Baseline (mm)	6.15 (0.65)	6.17 (0.63)	6.21 (0.68)	6.16 (0.70)
Peak dilation (mm)	0.29 (0.16)	0.28 (0.16)	0.36 (0.15)	0.33 (0.16)

*Standard deviations are presented between parentheses*.

An ANOVA on the peak dilation amplitude (Figures 2, 3, Table 2) with independent variables masker voice and spatial configuration showed a main effect of masker voice [*F*_(1, 23)_ = 5.40, *p* = 0.029], with larger pupil responses for the same-gender (female) masker than for the different-gender (male) masker. The effect of spatial configuration and the interaction effect between spatial configuration and masker voice were not statistically significant.

### Correlation analysis

Table 3 shows the results of the Spearman correlation analysis between RSpan, SicSpan, letter memory, TRT, and Trail-diff performances on the one hand, and the SRTs and pupil responses on the other hand.

**Table 3:** **Spearman correlation coefficients between text reception threshold (TRT), reading span, size comparison span (SicSpan), letter memory, trail making difference (Trail-diff), speech reception thresholds (SRTs), and the peak pupil dilation amplitude**.

	**SRTs**				**Peak dilation amplitude**			
	**M_0_**	**M_90_**	**F_0_**	**F_90_**	**M_0_**	**M_90_**	**F_0_**	**F_90_**
TRT	0.10	0.34	0.38	−0.05	−0.21	−0.03	0.06	0.19
Reading span	−0.34	0.06	0.03	−0.25	0.16	0.23	0.32	0.21
SicSpan	−0.07	*r* =-0.47 *p* = 0.021	−0.21	−0.28	0.37	0.16	−0.18	0.13
Letter memory	−0.04	−0.25	*r* =-0.53 *p* = 0.010	−0.15	0.25	0.29	−0.10	−0.04
Trail−diff	0.07	*r* = 0.64 *p* = 0.001	0.31	0.22	−0.27	−0.10	−0.08	−0.08

*Exact p-values are only provided for statistically significant (p < 0.05) correlation coefficients. Note that none of the correlation coefficients are statistically significant when controlling for multiple comparisons. M, male (different-gender) maskers; F, female (same-gender) maskers; 0, co-located maskers at 0°; 90, spatially separated maskers at ±90°*.

Higher SicSpan performance was associated with better (lower) SRTs in the condition with different-gender maskers and spatial separation. Better information updating ability (letter memory) was associated with lower (better) SRTs in the condition with same-gender maskers and no spatial separation. Finally, a larger Trail-dif score indicating poorer inhibition was associated with a higher (worse) SRT when different-gender maskers were presented with spatial separation. Note that none of the correlation coefficients are statistically significant when controlling for multiple comparisons (Bonferroni correction). Therefore, these correlation coefficients should be interpreted with caution. There were no statistically significant correlations between pupil response and cognitive variables.

The correlation analyses tentatively suggest that larger working memory capacity (SicSpan) and better control abilities (Trail-dif) are related to better speech perception when the masker voice is relatively dissimilar to the target voice (gender difference) and when spatial cues are available. In contrast, better information updating ability (letter memory) is associated with better speech perception when the masker voice is more similar to the target voice (same gender) and in the absence of spatial cues. Note that the results of the correlation analyses should be interpreted with caution due to the relatively small sample size.

## Discussion

In line with previous research (e.g., Brungart, 2001; Brungart et al., 2001; Neher et al., 2009, 2012), the current study showed that both spatial and voice cues help listeners to segregate target speech from distracter speech. The effect of spatial configuration was larger when target speech was masked with same-gender as compared to different-gender speech. Also, the effect of masker voice (same-gender vs. different-gender) was larger for co-located target and masker speech than for spatially separated target and masker speech. This pattern of results is in line with those observed for the LISN-S test (Cameron et al., 2011). Surprisingly, speech recognition performance was better for the same-gender as compared to the different-gender masker when masker speech was spatially separated. However, pupil responses were larger, indicating greater cognitive load for the same-gender as compared to the different gender maskers. This finding of better performance accompanied by greater cognitive load may be explained by the stronger temporal fluctuations of the female masker speech as compared to the male masker speech^1^. These stronger fluctuations allow more listening into the masker dips which may improve SRTs. These temporal fluctuations come into play when target and masker are spatially separated but are smeared out for the 0° condition where the two masking voice streams are co-located.

The pupil response data were only partly in line with the behavioral data. The peak pupil amplitude was larger when the masker and target voices were more similar (same-gender as compared to different-gender voices). No effect of spatial configuration on the pupil response was observed, indicating that although the availability of the spatial cues enhanced performance (i.e., lowered the SRTs), this benefit did not affect cognitive processing load during listening. A masker voice less similar to the target voice improved the SRTs and reduced the cognitive processing load as reflected by the pupil response, whereas adding spatial separation between the target and masker only resulted in an improvement in SRTs. The present data are in line with the results of Koelewijn et al. (2012a). In that study, target speech masked by interfering speech resulted in larger pupil responses than target speech masked by fluctuating noise. The average peak dilation amplitude observed in that study for female speech masked with a single male speech stream (0.32 mm for young listeners with normal hearing) was similar to that observed in the current study for the female 2-talker speech masker. In general, this suggests that the pupil response is larger when the masker characteristics are more similar to the characteristics of the target speech, whereas the physical spatial characteristics of the target and masker do not influence the pupil response. Although speech perception can be improved either by decreasing the target-masker similarity or by increasing the spatial separation of the target and masker, the concomitant cognitive load is reduced more by the reduction of target-masker similarity. One possible interpretation is that spatial separation eases speech understanding at a more peripheral level of processing, perhaps subcortical, whereas voice cues have to be dealt with at the cortical level by using top-down processing.

The current results are in line with previous data showing that factors that do have a large effect on the SRT (e.g., presenting stationary vs. fluctuating noise maskers) do not necessarily influence the pupil response during listening. In general, this study shows that the measurement of the pupil response adds information about the effects of masker characteristics on the speech recognition process that is not evident from inspection of the behavioral results alone. The results are relevant for future studies focusing on the influence of talker and masker location on speech perception performance and cognitive processing load in clinical populations (e.g., listeners with hearing impairment) and studies using other measures of cognitive processing load (e.g., see Gosselin and Gagné, 2011; Mackersie and Cones, 2011; Picou et al., 2011; Mishra et al., 2013a).

The SRT procedure converged on an SNR that corresponded to 50% sentence intelligibility; SNRs differed between the conditions. SNR differences are not likely to explain the condition effects on the pupil response as the SNR was highest in the condition with the largest pupil response. In listeners with normal hearing, higher SNRs result in smaller pupil responses if intelligibility is not controlled for (Zekveld et al., 2010). Together with the present data, previous pupillometric studies suggest that other stimulus characteristics, such as the similarity between masker and target stimulus, have a larger effect on the pupil dilation response than SNR has when intelligibility is kept constant (e.g., Koelewijn et al., 2012a).

It is important to note that the current results only included a very limited selection of conditions in terms of points on the psychometric function (around 50% intelligibility) and characteristics of the maskers and spatial configuration. The results may differ when other conditions (e.g., other spatial configurations, other and/or a different number of masker voices) are applied. However, the current results provide an example of how measures of cognitive processing load can complement behavioral measures in speech perception research.

Importantly, the differences in pupil response between conditions may have been attenuated by our selection of the baseline interval. The presentation of the masker 3 s prior to target speech onset revealed the difficulty level of the upcoming trial, as it indicated both the identity and the spatial origin of the masker speech. We applied a baseline correction on the pupil dilation response based on the average pupil size between 3 and 2 s prior to target speech onset (i.e., the first second of the presentation of the masker signal). In speech perception research, the baseline pupil size is usually determined in the 1 s prior to target speech onset (Zekveld et al., 2010; Kuchinsky et al., 2013). We used the pupil size in the first second of the masking stimulus instead as any influence of the knowledge of the masker type likely increased during the progression of interval with masker speech only. Listeners may anticipate the difficulty level of the upcoming sentence which is revealed by the identity and location of the masker. However, the information regarding the identity and spatial location of the masker was apparent right from the onset of the masker so this knowledge may still have affected the baseline pupil size, and hence the baseline-corrected peak pupil dilation amplitude. This is suggested by the higher baseline pupil size in the condition with same-gender maskers from the front as compared to the baseline pupil size in any of the other conditions (see Table 2).

Individual cognitive abilities were related to speech perception performance (SRTs) when no corrections for multiple comparisons were applied. Better SicSpan performance and better trail-making ability were associated with relatively low SRTs in the condition with different-gender maskers that were spatially separated from the target speech. In line with our hypotheses and Neher et al. (2009) and Glyde et al. (2013), this tentatively indicates that when it is relatively easy to distinguish the masker and target speech signals, larger working memory performance and better executive control were associated with better speech perception performance. In these conditions, individual differences in working memory capacity and executive function may come into play.

Better letter memory performance (information updating ability) was related to better SRTs in the condition with same-gender maskers with no spatial separation. We suggest that the cognitive load revealed by the pupil response may be related to demands on the ability to keep working memory updated with relevant information when few voice cues are available to segregate target speech from masker. As stated in the Results section, the results of the present correlation analysis should be interpreted with caution and require follow-up confirmatory research.

We have previously shown that better TRTs and SicSpan performances tend to be associated with larger pupil responses in the SRT test (Zekveld et al., 2011; Koelewijn et al., 2012b). In contrast, in the present study, none of the cognitive tests was related to the peak dilation amplitude of the pupil response. This difference between the current and past studies may be related to the characteristics of the participants. In Zekveld et al. (2011) and Koelewijn et al. (2012b), some of the participants were middle-aged. In other recent studies in which only young normal hearing listeners were included, the relation between cognitive abilities and the pupil response was only present when speech perception performance was very low (Zekveld and Kramer, 2014). Interestingly, in the present study, the pupil response was not related to cognitive abilities even in the conditions in which the performance (SRT) was related to one of the cognitive tests. This may suggest that even when good cognitive abilities improve speech recognition performance, they do not reduce the pupil response (cognitive processing load). This in turn may suggest that applying cognitive abilities to speech processing to achieve good speech recognition is no less effortful than achieving mediocre speech recognition without the assistance of good cognitive capacity. In general, the influence of inter-individual differences may affect the relation between task characteristics and the pupil response. Future studies should pull apart external and internal factors influencing the pupil response, for example by introducing individual differences as between-groups manipulation. It would also be interesting to apply other measures that may be related to cognitive processing load in such future studies. For example, Picou et al. (2011) showed an association between better performances on a complex working memory test and larger benefit from the availability of visual information (a recording of the face of the speaker) in word recognition (paired associates recall task) in noise. The authors interpret these data as reflecting that larger cognitive resource capacity allows listeners to use visual information for reducing cognitive processing load (cf. Mishra et al., 2013b).

In conclusion, differences between target and masker speech in terms of voice characteristics and spatial origin substantially enhance speech perception when speech is masked by interfering 2-talker babble. However, the same is not true of the pupil response. Performance is better and the pupil response is smaller when target and masker voices are of different gender than when they are of the same gender. On the other hand, although performance is better when target and masker are spatially separated, there is no significant difference in pupil response. This indicates that even when performance is improved by spatial separation cognitive processing load is not reduced. This demonstrates that measures reflecting cognitive processing load can add information about the speech perception process not provided by speech perception performance measures. This has implications for the design of future studies focusing on cognitive processing load during listening. The current findings indicate that the mechanisms that allow listeners to use voice characteristics and spatial information to segregate speech and masking speech are complex and affect the cognitive processing load required during listening.

### Conflict of interest statement

The authors declare that the research was conducted in the absence of any commercial or financial relationships that could be construed as a potential conflict of interest.

## Footnotes

^1^To obtain an impression of the speech modulation strengths, we analyzed 10 concatenated sentences of equal RMS for both the male and the female speaker by calculating the, 30-Hz low-pass filtered, Hilbert transforms of both signals. Next, we estimated spectral levels for the modulations of both speakers by calculating the average spectrum of the low-pass filtered Hilbert transforms. We found that the average spectrum of the female modulations was parallel to and 1.3 dB higher than that of the male speaker.
